# Consonantal Landmarks as Predictors of Dysarthria among English-Speaking Adults with Cerebral Palsy

**DOI:** 10.3390/brainsci11121550

**Published:** 2021-11-23

**Authors:** Chin-Ting Liu, Yuan-shan Chen

**Affiliations:** Department of Applied English, National Chin-Yi University of Technology, Taichung 411030, Taiwan

**Keywords:** cerebral palsy, dysarthria, landmark-based theory, consonants

## Abstract

The current study explored the possibility that the consonantal landmarks served as predictors of dysarthric speech produced by English-speaking adults with cerebral palsy (CP). Additionally, the relationship between the perceptual severity of dysarthric speech and the consonantal landmarks was explored. The analyses included 210 sentences from the TORGO database produced by seven English-speaking CP speakers with dysarthria and seven typically developing controls matched in age and gender. The results indicated that the clinical group produced more total landmark features than did the control group. A binominal regression analysis revealed that the improper control of laryngeal vibration and the inability to tactically control the energy in a voiced segment would lead to the higher likelihood of dysarthric speech. A multinominal regression analysis revealed that producing too many *+v* and *−v* landmark features would lead to higher perceptual severity levels among the CP speakers. Together with literature, the current study proposed that the landmark-based acoustic analysis could quantify the differences in consonantal productions between dysarthric and non-dysarthric speech and reflect the underlying speech motor deficits of the population in concern.

## 1. Introduction

Research on population-based studies around the world has reported that the prevalence estimates of individuals with cerebral palsy (CP) range from 2 to 3.9 per 1000 live births or children [1,2,3,4,5,6,7,8]. As CP can cause disturbances in cognitive development and motor disorders, dysarthria is one of the problems that CP speakers frequently suffer from in communication [9,10,11]. Therefore, research pertaining to CP speech has focused on a variety of linguistic aspects from a cross-linguistic perspective, including vowels, speaking rates, prosody, segmental error patterns, consonantal productions, literacy development, etc. (c.f., [10,12,13,14,15,16,17,18] among many others), with the aim of discovering the defining factors for those patients’ speech intelligibility. Results from the endeavor revealed that, among other critical factors, the quality of consonantal productions plays a significant role in the speech intelligibility of children and adults with CP [18,19,20,21,22,23,24,25,26].

Among the studies exploring the interplay between the consonantal productions and speech intelligibility of CP speakers with dysarthria, only a handful of studies have targeted English-speaking adults. Results from those studies uniformly demonstrated that the quality of fricative productions significantly contributes to speech intelligibility. For instance, Chen and Stevens [22] measured eight acoustical features of the fricative /s produced by eight CP speakers with dysarthria. Six of the acoustical features were reported to be highly correlated with those speakers’ overall speech intelligibility. Hernandez, Lee and Chung [26] acoustically analyzed eight English fricatives produced by ten dysarthric CP speakers. The results showed that CP speakers with lower intelligibility levels generally produced a longer fricative duration. On the other hand, studies encompassing a wider range of consonants in English showed that other consonantal categories also exerted large influence on speech intelligibility. In a multiple regression analysis, Ansel and Kent [19] showed that four (out of eight) acoustical parameters in their study could account for 79% of the variance in the intelligibility scores collected from the dysarthric CP speakers. Among the four variables, the fricative–affricate contrast in frication noise was the only consonantal variable. Other consonantal contrasts, such as nasality and voicing, could not statistically predict those speakers’ speech intelligibility. Similar findings were reported in Kim et al.’s [23] analysis based on listeners’ judgments. The results showed that the percentage of correctly articulated consonants decreased as the intelligibility levels decreased. More importantly, fricatives and affricates were associated with the highest error rates among dysarthric CP speakers.

As it is well established in the literature that consonantal productions are significant indicators of speech intelligibility among English-speaking CP adults with dysarthria, it is of critical importance to understand the consonantal production differences between dysarthric CP speakers and non-dysarthric typically developing (TD) speakers. Specifically, once the problematic features of consonantal productions from the dysarthric speakers are characterized and quantified, the underlying speech motor impairments and deficits can be identified, and this, in turn, can provide essential information for clinical specialists (e.g., language therapists, pediatricians, physiatrists and speech–language pathologists) in language rehabilitation and patients’ progress assessments. In this regard, the employment of acoustic analysis will be particularly helpful because it can reliably detect subtle differences in production [18,27,28], whereas traditional acoustic phonetic analysis required users to manually tag the boundaries of each of the sound segments in concern, which was a labor-intensive and time-consuming process [29,30]. Therefore, it is understandable that such a method is less likely to be employed by physiatrists and language therapists, as these clinicians are often constrained by tight schedules [31,32]. In short, although the significance of understanding the consonantal production features by dysarthric CP speakers has been recognized, the labor-intensive nature of acoustic analysis made it less feasible for practice. A semi-automatic and reliable tool that could be used to quantify and characterize the consonantal production features of the clinical group is in great need.

**Figure 1:**
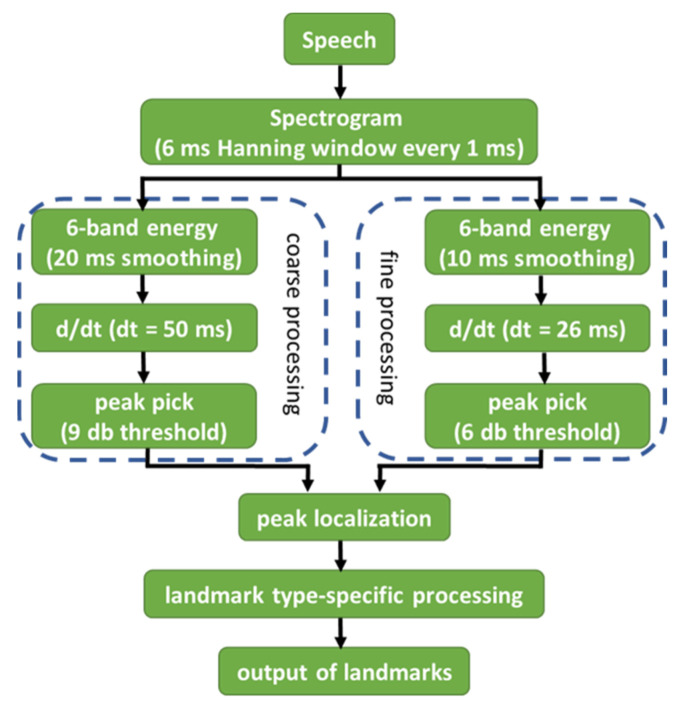
Flow diagram of the landmark detection algorithm (adopted from Liu [39] and Ishikawa and MacAuslan [43]).

In this connection, conducting landmark-based acoustic analysis by using the software SpeechMark© ([33], Boston, MA, USA) might be preferrable. The acoustic analysis was developed based on the landmark-based theory proposed by Stevens [34,35,36,37,38], Liu [39] and Howitt [40]. In the analysis, letters representing different acoustic landmarks and the positive/negative symbols showing the onset/offset of the sounds are presented when certain types of abrupt changes in spectrum or amplitude are detected. The theory hypothesizes that listeners rely on those abrupt changes in input to distinguish and recognize the heard speech sounds. The acoustic specifications and the associated articulatory interpretations of the six consonantal landmarks are shown in Table 1. Please note that the descriptions from Table 1 were directly adopted from MacAuslan [41], Ishikawa, MacAuslan and Boyce [42], and Liu [30], because these acoustic rules for generating the landmarks were specified by the software developers, and, hence, the resulting articulatory interpretations were constant as well. Based on Liu [39] and Ishikawa and MacAuslan [43], the landmark detection algorithm is shown in Figure 1. In general, the speech input was transformed into a spectrogram and was further processed with coarse smoothing (for suppressing too-brief events) and fine smoothing (for promoting higher-precision placement). The peaks in the signal represented the abrupt changes in speech input and the output of landmarks were determined based on the acoustic rules (for abrupt changes) specified in Table 1. By using the software SpeechMark©, the resulting patterns of the consonantal landmarks shown in Table 1 could be available within a reasonable period of time. Additionally, the underlying speech motor deficits of the speakers could be identified based on the articulatory interpretations in Table 1.

The *±g* and *±p* landmark features represent laryngeal-source events. The presence of the *+g* landmark indicates the onset of glottal vibration; the *−g* landmark indicates the offset of glottal vibration [39]. When the vibration sustains for at least 32 milliseconds, the *±p* landmark features are detected, which reflect the speaker’s ability to control the subglottal pressure and cricothyroid muscle [44]. The rest of the consonantal landmarks, *±b*, *±s*, *±f*, and *±v*, are categorized as vocal-tract events [41]. The *+b* landmark feature represents the bursts from an affricate or an aspirated stop [39]. It is detected when there is a silence interval followed by a 6 dB increase in high-frequency energy. The *−b* landmark feature is detected when there is a 6 dB decrease in high-frequency energy followed by a silence interval. As bursts occur within the region without glottal vibration, the *±b* landmarks are detected outside the region between a *+g* and a *−g*. The presence of the *−s* and *+s* landmark features signifies the closure and the release of a nasal or [l] [39]. When nasals and laterals are articulated, the constriction and the release of the vocal tract lead to the rapid decrease and increase in high-frequency energy bands. Therefore, the *±s* landmarks are identified when there are simultaneous power increases/decreases within a voiced segment (i.e., between *+g* and the next *−g*). The *±f* and *±v* landmark features are designated to detect unvoiced and voiced fricatives [42,45]. These landmarks capture the trait that fricatives have the highest frequencies in speech [46]. Therefore, the *+f* (for voiceless fricatives) and *+v* (for voiced fricatives) landmarks are detected when the power increases at high frequencies while the power decreases at low frequencies at the same time and vice versa for the *−v* and *−f* landmarks. In short, each of the six types of consonantal landmarks reflects the certain kinds of abrupt changes found in the acoustic signals and possesses unique and indicative articulatory interpretations.

Empirical studies using the software SpeechMark© have shown that the numbers of these acoustic landmarks could quantify the production traits of different populations and were highly correlated with the speech intelligibility of both typical and atypical populations. For instance, Ishikawa, MacAuslan and Boyce [42] analyzed the sentences produced by 36 English-speaking TD adults. The results indicated that female speakers produced more landmark features than did the male speakers. The authors further argued that more landmark features represented more contrast in the speech signal, giving rise to a higher speech intelligibility among female speakers. Ishikawa et al. [45] compared the number of consonantal landmarks between 33 English-speaking dysphonic adults and 36 TD controls. The results showed that the clinical group had more *±g*, *±b* and fewer *±s* landmark features, showing that there was insufficient voicing and more frequent interruptions in dysphonic speech. Additionally, the classification tree model indicated that the *+s* and *+b* landmarks were effective predictors for the dysphonic speech. Liu [30] analyzed the disyllabic words produced by 80 children ranging from four to seven years old. The results indicated that the younger age groups produced more *+b* landmarks than did the oldest age group. Furthermore, the results from the multiple regression analysis indicated that one unit increase in the *+b* landmark feature resulted in a 0.031 point decrease in those children’s speech intelligibility scores. Together with the findings from Ishikawa et al. [45], Liu [30] further proposed that too many and too few acoustic landmark features may equally reduce production quality and speech intelligibility. That is, it was not always a case of “the more, the better”. The quantity of the abrupt changes in spectrum or amplitude should be limited to a certain range, so that listeners can better comprehend the incoming speech signals. In short, by using the software SpeechMark©, the literature has clearly shown that the number of the consonantal landmarks could reflect the characteristics and quality of speakers’ consonantal productions.

Some studies have included landmark analysis in studying dysarthric speech secondary to CP, head trauma, or Parkinson’s Disease (PD); however only some selected landmark features were used in those studies. For instance, DiCicco and Patel [47] analyzed the sentences produced by six CP and four head trauma young males. The selected acoustic landmark features included *±g*, *±s*, and *±b*. The results showed that the participants frequently inserted unexpected landmarks in their productions and these additional acoustic cues might confuse listeners. The authors thus suggested “the utility of automatic landmark analysis in developing personalized dysarthria treatment” ([47], p. 213). Similarly, Boyce, Fell, Wilde and MacAuslan [48] included landmarks *±g*, *±s*, and *±b* in their analysis of 15 PD dysarthric speech. The results showed that the PD speakers produced fewer landmark clusters than did the controls, demonstrating those PD speakers’ lower levels of articulatory precision. Finally, Chenausky, MacAuslan and Goldhor [49] acoustically compared the speech productions from 12 TD and 10 PD speakers. Among other acoustic features, landmarks were used to determine the interval of voice onset time (VOT). The results indicated that the PD speakers had larger VOT variability than did the normal speakers. In short, the acoustic landmark analysis has been reported to be an effective index to dysarthric speech; however, as the relevant literature focused on a subset of the consonantal landmarks, the current study intended to include all six consonantal landmarks to provide a more comprehensive picture.

In summary, acoustic analysis revealed that the quality of the consonantal productions exerted direct influence on the speech intelligibility of English-speaking CP adults with dysarthria. Therefore, it is significant to quantify and characterize the consonantal production features of dysarthric and non-dysarthric speakers so that the underlying speech motor deficits of the CP speakers can be identified. Although acoustic analysis might be a promising tool, due to the fact that traditional acoustic analysis was labor-intensive and time-consuming, the method was less likely to be systematically employed. In this connection, the landmark-based acoustic analysis could be a preferrable option because, with the software SpeechMark©, the analysis could be completed within a reasonable time span. Additionally, empirical studies have repeatedly demonstrated that the acoustic landmarks (i.e., the abrupt changes in spectrum or amplitude) detected from speech input could reflect the consonantal production features of a wide range of populations.

Therefore, the purpose of this study was to explore the differences of the consonantal productions from English-speaking CP adults with and without dysarthria by using the landmark-based acoustic analysis. Furthermore, the relationship between the perceptual severity of dysarthric speech and the consonantal landmarks was explored. It is expected that the numbers of landmarks produced by CP speakers and those produced by TD controls would be different, which could reflect the quality and the underlying speech motor deficits of the clinical group. In addition, by using regression analyses, the unique contribution of the landmarks to dysarthric speech could be identified. The resulting patterns could serve as essential references for physiatrists and language therapists in progress assessment for CP individuals with dysarthria and could help them to efficiently and reliably identify the underlying speech motor impairments of their patients.

## 2. Methods

### 2.1. Speech Samples

The data included in the current study were from the TORGO database [50,51,52], which was co-established by the departments of Computer Science and Speech-Language Pathology at the University of Toronto and the Holland Bloorview Kids Rehabilitation hospital. In order to explore the underlying articulatory parameters of speech production and hence to develop advanced models in automatic speech recognition for dysarthric speakers, the database included not only the sound files of individuals with speech disability, but also 2D and 3D articulatory features from the speakers. Specifically, the database contained speech samples from three female and four male English-speaking CP adults (including spastic, athetoid, and ataxic diagnosis) with dysarthria as well as one male English-speaking dysarthric speaker whose dysarthria resulted from amyotrophic lateral sclerosis. The ages of the dysarthric speakers ranged from 16 to 50 years old ([51]). The speech samples reported in the current study were from the seven CP speakers with dysarthria in the TORGO database. According to Rudzicz, Namasivayam and Wolff [52], a pre-visit questionnaire was administrated to ensure that the cognitive function of the CP speakers was above or at level VIII on the Rancho scale [53]. The participants must not have had a history of substance abuse, severe hearing or visual problems. In addition, the reading ability of the participants must have been at least at a 6th grade elementary level. The database also provided data from seven TD controls matched in age and gender, which were also included in the current analysis.

Fifteen sentences from the restricted-sentence section in the database were selected because the sound recordings of those sentences were available among the 14 speakers. These sentences were phoneme-rich and were frequently used in speech intelligibility tests or in assessing the perceptual features of connected speech in dysarthric speakers (c.f., [53,54,55,56,57]). The 15 sentences included in the analysis are listed in (1).

(1) a. Except in the winter when the ooze or snow or ice prevents.
   b. He slowly takes a short walk in the open air each day.
   c. Usually minus several buttons.
   d. You wished to know all about my grandfather.
   e. But he always answers, banana oil.
   f. The quick brown fox jumps over the lazy dog.
   g. She had your dark suit in greasy wash water all year.
   h. Giving those who observe him a pronounced feeling of the utmost respect.
   i. We have often urged him to walk more and smoke less.
   j. A long, flowing beard clings to his chin.
   k. Yet he still thinks as swiftly as ever.
   l. Well, he is nearly ninety-three years old.
   m. He dresses himself in an ancient black frock coat.
   n. Grandfather likes to be modern in his language.
   o. When he speaks, his voice is just a bit cracked and quivers a trifle.

The speech productions were recorded by two microphones. In the current analysis, sound files recorded by the head-mounted electret microphone were primarily used for analysis. In the rare cases that the sound files recorded with the electret microphone were not available, the sound files recorded by using an Acoustic Magic Voice Tracker array microphone were used.

### 2.2. Landmark-based Acoustic Analysis and Perceptual Analysis

The authors of the study analyzed all 210 sentences (i.e., 14 participants * 15 sentences) produced by the participants by using the software SpeechMark© (WaveSurfer Plug-in, Windows Edition, Version 1.0.39). The “female” and “male” options in SpeechMark© were used in accordance with the gender of the participants so that the fundamental frequency in the analysis could be adjusted. A custom-written program was used to automatically compute the total number of each landmark type in the output. In order for the results to be comparable for future studies and to be employed in clinical settings, the number of each landmark type per syllable was calculated for each of the sentences produced by each participant. For instance, there were 15 syllables in (1a). When there were 17 *+p* landmarks detected in the sentence produced by a participant, the number of the *+p* landmark per syllable for the sentence would be 1.133 (i.e., 17/15).

The perceptual analysis was based on the percentage of consonants correct (PCC) score developed by Shriberg and Kwiatkowski [58]. PCC has been widely adopted in language disorder studies and has been reported to highly correlate with speech intelligibility [59,60]. A licensed language therapist was invited to evaluate the PCC score of all the sentences produced by the CP speakers. To establish the inter-rater reliability, a native speaker of English was also invited to evaluate all the sentences produced by the first three participants in the list (i.e., around 43% of the total sentences). The numbers of the correctly produced consonants from these two raters were used for Pearson’s correlation and the resulting *r* was 0.867 (*p* < 0.001). The severity of involvement of the individual sentence was determined based on the PCC. According to Shriberg and Kwiatkowski [58], the severity level was *Mild*, *Mild–Moderate*, *Moderate–Severe* and *Severe* when the resulting PCC score was between 85–100%, 65–84%, 50–64% and 0–49%, respectively. The individual participant’s severity level was determined by averaging the PCC scores from the 15 sentences he/she produced. Although the severity cutoff scores from Shriberg and Kwiatkowski [58] were originally determined based on children, the criteria were also adopted for studies focusing on adults [61,62,63]. Irrespective of the participants’ chronological ages, the percentages of the correctly pronounced consonants did reflect the individual participant’s severity level in speech production and, therefore, the current study followed the literature pertaining to adult language disorders (c.f., [61,62,63]) and adopted the cutoff scores from Shriberg and Kwiatkowski [58].

### 2.3. Descriptive and Inferential Statistics

First, an independent-samples t-test was used to investigate whether the differences in the total landmarks per syllable between dysarthric speech and normal speech were statistically significant. Second, in order to understand the variability across individual speakers and sentences, the resulting landmark patterns of each speaker and each sentence were presented. Next, a binomial logistic regression was performed to look at the effects of each landmark type (per syllable) and gender on the likelihood that the sentence would be categorized as normal or dysarthric speech. Finally, a multinomial logistic regression was used to explore the relationship between the acoustic landmarks and the PCC severity levels among CP speakers.

## 3. Results

The results of the landmark-based acoustic analysis are shown in Table 2. The analysis revealed that there was an average of 7.61 landmarks (SD = 4.46) in each of the sentences produced by the CP speakers while there was an average of 5.719 landmarks (SD = 1.493) in each of the sentences produced by the TD speakers. An independent-samples t-test was performed to investigate whether the differences between the average numbers of the landmark per sentence were statistically significant. As Levene’s test for equality of variances was not assumed, the more conservative statistics were reported here. The results revealed that the differences were statistically significant, *t* (127.001) = 4.119, *p* < 0.001. That is, there were generally more landmarks found in the sentences produced by the CP speakers.

In order to understand the extent of variability among the CP individuals with dysarthria, the individual speaker results for the different landmarks, average PCC scores and the resulting severity levels are presented in Table 3. The individual speakers’ acoustic landmarks among the TD speakers are presented in Table 4 for reference.

The average landmarks per sentence ranged from 4.718 to 16.288 among the CP speakers while the numbers ranged from 5.125 to 6.163 among the TD counterparts. Among the CP speakers, producing too many and too few total landmarks would result in higher PCC severity levels (c.f., CP6 and CP1, respectively). The high variability of the resulting acoustic landmark patterns among the clinical group might have resulted from the high variability of the severity levels among the CP speakers.

The resulting landmark patterns were analyzed in order to explore whether they differed among different sentences, and Table 5 presents the results from the analysis of individual sentences. The average PCC scores for each of the 15 sentences produced by the CP speakers were also presented. For each of the 15 sentences, the total landmarks from CP speakers were higher than those from the TD controls. That is, irrespective of the contents of the sentences, CP speakers produced a larger number of landmark features. The average PCC scores among the CP speakers ranged from 67.28% to 84.13%.

A binomial logistic regression was performed to explore the effects of each landmark type (per syllable) and gender on the likelihood that the sentence would be categorized as normal or dysarthric speech. The logistic regression model was statistically significant, *χ^2^* (12) = 88.352, *p* < 0.001. The model explained 45.8% (Nagelkerke *R^2^*) of the variance in the dysarthric/normal speech and correctly classified 79% of the individual sentences. The variables in the equation are shown in Table 6. The results revealed that having more *+g*, *+p* and *−s* landmarks per syllable as well as having fewer *−g*, *−p* and *+v* landmarks per syllable had a positive effect on the likelihood of producing dysarthric speech; however, gender was not a statistically significant predictor.

An additional multinominal logistic regression was performed to explore the relationship between acoustic landmark features and the PCC severity levels of each of the sentences produced by the CP speakers. The first category (i.e., Mild) was selected as the reference category. The results indicated that the logistic regression model was statistically significant, *χ^2^* (36) = 88.352, *p* = 0.001. The landmark features *+v* and −*v* were two significant predictors in the model (*p* = 0.021 for *+v* and *p* < 0.001 for *−v*). The parameter estimates further revealed that one unit increase in the *+v* landmark feature among sentences with a PCC score between 65–84% (i.e., Mild–Moderate) would decrease the possibility that the sentences would be categorized as Mild. Additionally, one unit increase in the *−v* landmark feature among sentences with a PCC score between 50–64% and between 0–49% (i.e., Moderate–Severe and Severe) would decrease the possibility that the sentences would be categorized as Mild. In short, the higher numbers of the *+v* and *−v* landmark features among CP speakers would lead to the higher likelihood that their speech would be categorized with higher severity levels.

## 4. Discussion

The purpose of this study was to employ the landmark-based acoustic analysis to examine the differences of the consonantal productions produced by English-speaking CP adults with dysarthria and those produced by TD controls. Additionally, the relationship between the acoustic landmark features and the language therapist’s perceptions of the dysarthric speech was explored. Results from a total of 210 sentence productions revealed that CP speakers with dysarthria generally produced more landmarks per sentence. In addition, irrespective of the contents and the length of the sentences, the CP speakers uniformly produced a higher number of average landmarks per syllable/sentence. Results from the binominal logistic regression indicated that 45.8% of the variance in the dysarthric and normal speech could be accounted for by gender and the landmark-based acoustic analysis; 79% of the individual sentences were correctly classified. The results also revealed that producing more *+g*, *+p* and *−s* landmarks per syllable as well as fewer *−g*, *−p* and *+v* landmarks per syllable would increase the likelihood of generating dysarthric sentences. Results from the multinominal logistic regression revealed that the higher numbers of the *±v* landmark features among CP speakers would lead to the higher possibility that their productions would be categorized with higher severity levels. Based on the obtained results, several issues are discussed below.

The *±g* and the *±p* landmarks were two landmark types that could effectively predict the dysarthric speech. According to Table 1, both types of landmarks reflected the laryngeal motions. A *+g*/−*g* landmark was detected when there was an onset/offset of vocal folds free vibration. When the vibration was sustained for at least 32 milliseconds, a *+p*/*−p* landmark was detected at the onset/offset. The fact that having more *+g* and *+p* landmarks would lead to the higher likelihood of dysarthric speech showed that those CP speakers were not able to properly control their laryngeal motions, giving rise to too many onset vocal folds’ free vibrations. Similarly, the lower numbers of the *−g* and *−p* landmarks indicated that the CP speakers’ inability to maintain the laryngeal gestures at the offset of the production would lead to a higher possibility of producing dysarthric sentences. That is, the results from the landmark-based analysis revealed that too many onset laryngeal motions and insufficient offset laryngeal motions resulted in the speech motor deficits in dysarthric speech. Other important indices to the dysarthric speech were the excessive number of the *−s* landmarks and the relatively lower number of the *+v* landmarks. The *−s* landmark represented the closure of nasal or lateral segments and were detected in a voiced segment (i.e., between *+g* and the next *−g* landmarks). The *+v* landmark was also detected in a voiced segment when there were 6 dB increases at high frequencies, representing the onset of a voiced fricative. Therefore, the results indicated that the essential differences between normal and dysarthric speech were the excessive number of abrupt decreases in the acoustic energy as well as the fewer number of abrupt increases at high frequencies among voiced segments, which collaboratively reflected the inferior speech motor control ability of CP individuals. That is, they failed to tactically control the energy in the voiced segments so that decreases in acoustic energy were frequently detected while increases in acoustic energy could not be reliably detected. To sum up, the current findings revealed two major underlying motor deficits among the dysarthric speakers. First, those speakers lacked the speech motor control ability to maintain the finer-grained laryngeal activity. Second, they were unable to properly control the articulatory gestures in a voiced segment.

Results from the multinominal logistic regression analysis further revealed the relationship between the perceptual severity levels of the productions and the acoustic landmark features among the CP speakers. The number of the *±v* landmark features had a negative correlation with the perceived severity levels of the dysarthric speech. That is, when the number of the *±v* landmark features increased, the perceived severity level of the dysarthric speech became worse. Based on Table 1, the *±v* landmark features were detected when there were 6 dB power increases or decreases at high frequencies of a voiced segment and were corresponding to the onset or the offset of a voiced fricative. These resulting patterns were in line with the order of segmental acquisition predicted by Kent [64], who proposed that producing fricatives required finer-grained speech motor control ability and would take a longer time to master. Taken together, the results from the perceptual and acoustic landmark analyses indicated that the inability to tactically produce voiced fricatives would lead to higher levels of perceptual severity among the CP speakers.

The landmark-based acoustic analysis could reflect the population-specific articulatory difficulties that influenced the quality of speech. For instance, Liu [30] found that the increase in the number of the *+b* landmark feature among age four-to-seven Mandarin-acquiring children resulted in the decrease of the intelligibility score. This showed that those who had a lower speech intelligibility score generally generated too many obstruent bursts. Ishikawa et al. [45] found that dysphonic speech contained an excessive number of the *+b* landmark features and an insufficient number of the *+s* landmark features. This showed that, in addition to the issue of the obstruent bursts, the clinical group in the study was not able to properly formulate the articulatory gesture for producing a nasal or a lateral. Unlike the obstruent bursts issue found in the Mandarin-acquiring children and English-speaking dysphonic individuals, the current findings indicated that the laryngeal vibration, manifested by the *±g* and *±p* landmarks, were the primary difficulties for English-speaking CP speakers with dysarthria. Additionally, the number of the *±v* landmark features were negatively correlated with the perceptual speech severity levels among the CP speakers. In short, the landmark-based acoustic analysis could reflect population-specific articulatory difficulties. 

This study contributes to the existing literature on analyzing dysarthric speech with consonantal acoustic landmarks. As the current version of acoustic landmark analysis was not previously developed, earlier studies focusing on dysarthric speech secondary to CP, head trauma, or PD did not include all six consonantal landmarks in the analysis. Rather, those studies reported results from two [49] or three [47,48] of the landmark types. The inclusion of the laryngeal-source related landmark features *±p* and the vocal-tract related landmark features *±v* in the current study revealed additional facets of difficulties that the CP speakers encountered. Specifically, while the *±g* landmark features revealed that the CP speakers had issues in generating vocal fold free variations, the inclusion of the landmark features *±p* further revealed that those CP speakers had difficulties in maintaining the vibration once it had been initiated. Furthermore, the current studies also revealed that the higher numbers of the *±v* landmark features led to their higher levels of severity involvement. In short, the current study not only confirmed DiCicco and Patel’s view [47] that the landmark analysis could “relate acoustic-phonetic events to underlying articulatory behavior” (p. 216), but also demonstrated that the inclusion of the complete set of landmark features would reveal the multifaceted features of dysarthric speech.

The landmark-based acoustic analysis also bears significant values for empirical studies and clinical applications. Although the imprecise consonantal productions have been widely recognized as a hallmark of dysarthria [65,66,67], the diversity of the acoustic properties among consonants has been identified as a challenge in acoustic measurements (c.f., [66,68]). This might explain why only selected acoustic features were included in literature investigating the relationship between consonants and perceptual analyses among adult speakers with dysarthria. With the application of the landmark-based acoustic analysis, multiple acoustic features of consonants could be analyzed at once and the resulting patterns provided a more holistic view. Furthermore, although the current study targeted adult dysarthric speech secondary to CP, the landmark-based acoustic analysis could also be applied to pediatric CP patients for assessment and intervention purposes. As early diagnosis of dysarthria for children with CP and early intervention for their speech productions are an essential clinical endeavor [10,69], the application of the landmark-based acoustic analysis could provide the holistic picture of the deficits in consonantal productions among CP children with high risks of dysarthria. In addition, the resulting patterns of the landmark-based acoustic analysis could serve as the indices for intervention evaluations and progresses of CP children’s consonantal development.

The current findings were not without limitations, and the limitations could become the directions for future research. First, the current model from the binominal logistic regression analysis could only explain 45.8% of the variance in the dysarthric/normal speech. Other factors could be included in future analyses so that a larger portion of the variance could be accounted for. For instance, acoustic parameters pertaining to vowels could potentially be included, as previous studies have shown that several acoustic properties of vowels would influence the quality of dysarthric speech (e.g., [70,71]). Next, detailed information regarding individual participants’ ages was not available in the TORGO ’database, and, therefore, the effects of the participants’ ages on dysarthric speech required further exploration. Specifically, it has been shown by Kuschmann and Brenk [72] that the critical acoustic parameters distinguishing young CP speakers from the TD controls varied in accordance with the ages of the participants. Therefore, future studies might wish to include the participants’ ages as a variable to investigate if the critical landmark futures differentiating the clinical group from the control group would vary in accordance with the ages, too. Finally, all the sentences included in the analysis (c.f., (1) and Table 5) revealed the same resulting landmark patterns. That is, the CP speakers generally produced a higher number of total landmarks irrespective of the sentence contents and length. One potential reason for the observed phenomenon was that those sentences were phoneme-rich sentences and had been selected for the purpose of clinical settings. Therefore, one important direction for future endeavors is to explore the landmark differences between sentence recitation and spontaneous productions. That is, if the abrupt changes in spectrum or amplitude are the key signals for speech perceptions, as proposed by the landmark theory, it is expected that the resulting landmark patterns (i.e., average landmarks per syllable) would be similar to those observed in the current study. Investigations in this direction would strengthen the vali-dation of the acoustic landmark analysis as a tool for clinical assessment.

## 5. Conclusions

By using the landmark-based acoustic analysis, the current study investigated the quality of the consonantal productions from English-speaking CP adults with dysarthria. A total of 210 sentences produced by seven CP adults with dysarthria, and seven age- and gender-matched TD controls were collected from the TORGO database [50,51,52]. The results indicated that the clinical group produced a higher number of total landmark features than did the control group. The binominal logistic regression indicated that generating more *+g*, *+p* and *−s* landmarks per syllable as well as producing fewer *−g*, *−p* and *+v* landmarks per syllable, led to the higher possibility of producing dysarthric speech. Based on the articulatory interpretations of the landmark features, the resulting patterns revealed that issues in sustaining laryngeal vibration as well as in coordinating the energy in the voiced segments would lead to the higher likelihood of generating dysarthric speech. The multinominal logistic regression revealed a negative correlation between the number of the *±v* landmark features and the perceptual severity of the production. The acoustic landmark analysis was argued to be one feasible tool in understanding the underlying speech motor deficits of CP speakers with dysarthria. It was hoped that future studies targeting a less heterogeneous CP group and spontaneous speech would enhance the validation of the landmark-based analysis at clinical settings.

## Figures and Tables

**Table 1:** Acoustic rules and articulatory interpretations of the six abrupt-consonantal landmarks (adopted from MacAuslan [41], Ishikawa, MacAuslan and Boyce [42], and Liu [30]).

Symbol	Mnemonic	Acoustic Rule	Articulatory Interpretation
*±g*	Glottal	Beginning/end of sustained laryngeal vibration/motion	Onset/offset of vocal folds’ free vibration
*±p*	Periodicity	Beginning/end of sustained periodicity (syllabicity) lasting for at least 32 milliseconds	The presence of *±p* reflects the speaker’s ability to properly control the subglottal pressure and cricothyroid muscle.
*±b*	Burst	At least 3 of 5 frequency bands show simultaneous power increases/decreases of at least 6 dB in both the finely smoothed and the coarsely smoothed contours, in an unvoiced segment (not between *+g* and the next *−g*)	Presence of a fricative, affricate or aspirated stop burst consonant (i.e. *+b*) or cessation of frication or aspiration noise (i.e. *−b*)
*±s*	Syllabic	At least 3 of 5 frequency bands show simultaneous power increases/decreases of at least 6 dB in both the finely smoothed and the coarsely smoothed contours, in a voiced segment (between *+g* and the next *−g*)	Closure or release of a nasal or /l/
*±f*	Unvoiced frication	At least 3 of 5 frequency bands show simultaneous 6 dB power increases/decreases at high frequencies and decreases/increases at low frequencies (unvoiced segment)	Onset/offset of an unvoiced fricative
*±v*	Voiced frication	At least 3 of 5 frequency bands show simultaneous 6 dB power increases/decreases at high frequencies and decreases/increases at low frequencies (voiced segment)	Onset/offset of a voiced fricative

**Table 2:** Mean and standard deviation (in parentheses) of the average landmark features per syllable/sentence.

Landmark Features	Dysarthric Speech	Normal Speech
***+g***	0.964 (0.463)	0.809 (0.241)
***−g***	0.961 (0.462)	0.827 (0.248)
***+p***	2.008 (1.75)	1.341 (0.462)
***−p***	1.622 (1.252)	1.195 (0.389)
***+b***	0.394 (0.214)	0.338 (0.206)
***−b***	0.127 (0.114)	0.169 (0.12)
***+s***	0.7 (0.399)	0.482 (0.27)
***−s***	0.752 (0.439)	0.43 (0.246)
***+f***	0.003 (0.025)	0.013 (0.04)
***−f***	0.01 (0.028)	0.029 (0.048)
***+v***	0.023 (0.055)	0.049 (0.085)
***−v***	0.045 (0.078)	0.038 (0.056)
**Total Landmarks** **per Sentence**	7.61 (4.46)	5.719 (1.493)

**Table 3:** CP individual dysarthric speaker’s mean and standard deviation (in parentheses) of the average landmark features per syllable/sentence.

Landmark Features	CP 1 (Female)	CP 2 (Female)	CP 3 (Female)	CP 4 (Male)	CP 5 (Male)	CP 6 (Male)	CP 7 (Male)
***+g***	0.680 (0.246)	0.789 (0.225)	0.761 (0.222)	0.807 (0.237)	0.783 (0.242)	1.631 (0.629)	1.295 (0.320)
***−g***	0.686 (0.243)	0.796 (0.227)	0.744 (0.226)	0.801 (0.245)	0.783 (0.242)	1.624 (0.626)	1.295 (0.320)
***+p***	0.941 (0.431)	1.154 (0.343)	1.392 (0.473)	1.530 (0.447)	1.163 (0.391)	5.568 (2.100)	2.307 (0.597)
***−p***	0.835 (0.354)	0.969 (0.270)	1.149 (0.359)	1.344 (0.427)	1.017 (0.335)	4.141 (1.444)	1.901 (0.461)
***+b***	0.378 (0.152)	0.410 (0.231)	0.279 (0.173)	0.358 (0.140)	0.454 (0.163)	0.284 (0.221)	0.598 (0.244)
***−b***	0.155 (0.146)	0.113 (0.070)	0.076 (0.076)	0.064 (0.068)	0.203 (0.154)	0.096 (0.096)	0.184 (0.091)
***+s***	0.510 (0.259)	0.395 (0.170)	0.736 (0.210)	0.809 (0.297)	0.306 (0.136)	1.394 (0.252)	0.751 (0.182)
***−s***	0.510 (0.160)	0.417 (0.173)	0.693 (0.240)	0.967 (0.262)	0.559 (0.236)	1.484 (0.464)	0.634 (0.338)
***+f***	0.000 (0.000)	0.000 (0.000)	0.010 (0.040)	0.000 (0.000)	0.000 (0.000)	0.000 (0.000)	0.013 (0.052)
***−f***	0.000 (0.000)	0.007 (0.026)	0.004 (0.016)	0.004 (0.016)	0.023 (0.041)	0.005 (0.020)	0.024 (0.042)
***+v***	0.018 (0.038)	0.041 (0.064)	0.074 (0.093)	0.000 (0.000)	0.026 (0.058)	0.000 (0.000)	0.005 (0.020)
***−v***	0.005 (0.020)	0.055 (0.096)	0.074 (0.067)	0.019 (0.046)	0.047 (0.059)	0.061 (0.125)	0.050 (0.075)
**Total Landmarks** **per Sentence**	4.718 (1.736)	5.145 (1.193)	5.993 (1.437)	6.703 (1.535)	5.365 (1.437)	16.288 (5.034)	9.058 (2.011)
**Average PCC Score**	44.32% (12.23)	94.85% (4.12)	100% (0)	65.24% (15.41)	76.14% (10.21)	58.77% (11.87)	81.12% (8.98)
**Severity Level**	*Severe*	*Mild*	*Mild*	*Mild–Moderate*	*Mild–Moderate*	*Moderate–Severe*	*Mild*

**Table 4:** TD individual speaker’s mean and standard deviation (in parentheses) of the average landmark features per syllable/sentence.

Landmark Features	TD 1 (Female)	TD 2 (Female)	TD 3 (Female)	TD 4 (Male)	TD 5 (Male)	TD 6 (Male)	TD 7 (Male)
***+g***	0.883 (0.217)	0.715 (0.238)	0.854 (0.219)	0.884 (0.227)	0.802 (0.216)	0.938 (0.232)	0.589 (0.187)
***−g***	0.922 (0.233)	0.738 (0.248)	0.879 (0.231)	0.888 (0.238)	0.812 (0.222)	0.942 (0.237)	0.608 (0.190)
***+p***	1.371 (0.467)	1.392 (0.554)	1.387 (0.386)	1.299 (0.464)	1.389 (0.449)	1.513 (0.536)	1.032 (0.267)
***−p***	1.220 (0.397)	1.171 (0.404)	1.133 (0.303)	1.213 (0.414)	1.283 (0.406)	1.408 (0.445)	0.940 (0.211)
***+b***	0.323 (0.173)	0.264 (0.204)	0.425 (0.204)	0.352 (0.209)	0.322 (0.212)	0.365 (0.244)	0.316 (0.195)
***−b***	0.134 (0.125)	0.085 (0.058)	0.193 (0.102)	0.205 (0.105)	0.211 (0.143)	0.167 (0.108)	0.189 (0.143)
***+s***	0.481 (0.266)	0.705 (0.195)	0.544 (0.201)	0.568 (0.228)	0.205 (0.144)	0.201 (0.147)	0.667 (0.166)
***−s***	0.402 (0.160)	0.610 (0.278)	0.555 (0.184)	0.407 (0.229)	0.233 (0.173)	0.196 (0.108)	0.608 (0.179)
***+f***	0.010 (0.026)	0.000 (0.000)	0.015 (0.040)	0.013 (0.052)	0.032 (0.071)	0.015 (0.031)	0.006 (0.022)
***−f***	0.042 (0.054)	0.009 (0.025)	0.035 (0.057)	0.025 (0.044)	0.060 (0.056)	0.010 (0.027)	0.023 (0.053)
***+v***	0.044 (0.068)	0.047 (0.069)	0.100 (0.092)	0.021 (0.036)	0.022 (0.039)	0.004 (0.017)	0.101 (0.151)
***−v***	0.050 (0.07)	0.046 (0.052)	0.042 (0.070)	0.021 (0.038)	0.028 (0.041)	0.030 (0.059)	0.046 (0.058)
**Total Landmarks** **per Sentence**	5.882 (1.576)	5.784 (1.791)	6.163 (1.349)	5.896 (1.552)	5.399 (1.458)	5.787 (1.661)	5.125 (0.987)

**Table 5:** Mean and standard deviation (in parentheses) of the average landmark features per syllable among individual sentences in (1).

Landmarks	(1a)		(1b)		(1c)		(1d)		(1e)		(1f)		(1g)		(1h)		(1i)		(1j)		(1k)		(1l)		(1m)		(1n)		(1o)	
	CP	TD	CP	TD	CP	TD	CP	TD	CP	TD	CP	TD	CP	TD	CP	TD	CP	TD	CP	TD	CP	TD	CP	TD	CP	TD	CP	TD	CP	TD
***+g***	0.933	0.952	1.102	0.837	0.558	0.494	0.727	0.727	0.714	0.743	1.078	0.935	1.121	0.769	0.952	0.738	1.060	0.905	0.937	0.841	1.114	1.086	0.771	0.529	1.167	1.060	0.917	0.571	1.304	0.955
	(0.353)	(0.254)	(0.412)	(0.239)	(0.254)	(0.156)	(0.283)	(0.223)	(0.248)	(0.237)	(0.338)	(0.194)	(0.796)	(0.172)	(0.432)	(0.127)	(0.438)	(0.122)	(0.326)	(0.108)	(0.478)	(0.168)	(0.373)	(0.125)	(0.433)	(0.165)	(0.640)	(0.155)	(0.597)	(0.112)
***−g***	0.933	0.962	1.112	0.837	0.558	0.494	0.740	0.753	0.686	0.729	1.078	0.935	1.110	0.769	0.944	0.770	1.060	0.940	0.937	0.841	1.100	1.086	0.771	0.557	1.167	1.107	0.917	0.607	1.304	1.018
	(0.353)	(0.234)	(0.436)	(0.239)	(0.254)	(0.156)	(0.275)	(0.245)	(0.254)	(0.236)	(0.338)	(0.194)	(0.768)	(0.172)	(0.437)	(0.141)	(0.438)	(0.142)	(0.326)	(0.108)	(0.480)	(0.212)	(0.373)	(0.172)	(0.433)	(0.185)	(0.640)	(0.142)	(0.597)	(0.107)
***+p***	2.457	1.695	2.286	1.510	1.104	0.597	1.468	1.195	1.300	1.243	1.883	1.506	2.703	1.209	1.897	1.270	2.262	1.476	1.730	1.302	2.100	1.743	1.743	0.900	2.464	1.655	1.714	1.083	3.009	1.723
	(2.104)	(0.525)	(1.893)	(0.281)	(0.920)	(0.240)	(1.144)	(0.308)	(0.744)	(0.538)	(1.116)	(0.442)	(2.771)	(0.280)	(1.531)	(0.380)	(1.523)	(0.214)	(1.368)	(0.543)	(1.127)	(0.395)	(1.669)	(0.351)	(2.343)	(0.286)	(1.848)	(0.167)	(3.057)	(0.231)
***−p***	1.876	1.476	1.755	1.255	0.961	0.584	1.195	1.013	1.014	1.157	1.623	1.403	2.066	1.176	1.587	1.159	1.810	1.310	1.476	1.159	1.814	1.500	1.314	0.829	2.024	1.452	1.417	0.964	2.402	1.491
	(1.476)	(0.451)	(1.150)	(0.294)	(0.776)	(0.277)	(0.863)	(0.143)	(0.511)	(0.391)	(0.809)	(0.399)	(1.792)	(0.250)	(1.166)	(0.342)	(1.154)	(0.191)	(0.999)	(0.425)	(0.838)	(0.342)	(1.128)	(0.298)	(1.686)	(0.276)	(1.399)	(0.203)	(2.252)	(0.235)
***+b***	0.371	0.267	0.510	0.378	0.195	0.156	0.364	0.325	0.314	0.229	0.519	0.545	0.264	0.264	0.333	0.246	0.500	0.298	0.444	0.667	0.486	0.500	0.200	0.114	0.524	0.440	0.417	0.250	0.473	0.393
	(0.167)	(0.168)	(0.133)	(0.107)	(0.133)	(0.180)	(0.117)	(0.147)	(0.285)	(0.125)	(0.281)	(0.148)	(0.132)	(0.124)	(0.111)	(0.090)	(0.204)	(0.116)	(0.308)	(0.192)	(0.219)	(0.300)	(0.208)	(0.069)	(0.178)	(0.185)	(0.204)	(0.108)	(0.107)	(0.148)
***−b***	0.057	0.114	0.102	0.092	0.091	0.117	0.104	0.156	0.086	0.129	0.234	0.221	0.132	0.099	0.103	0.175	0.083	0.167	0.175	0.254	0.186	0.186	0.057	0.086	0.143	0.286	0.190	0.190	0.170	0.268
	(0.060)	(0.063)	(0.081)	(0.068)	(0.129)	(0.101)	(0.063)	(0.069)	(0.107)	(0.095)	(0.137)	(0.072)	(0.123)	(0.131)	(0.059)	(0.093)	(0.083)	(0.136)	(0.126)	(0.178)	(0.177)	(0.121)	(0.079)	(0.069)	(0.079)	(0.126)	(0.142)	(0.115)	(0.112)	(0.129)
***+s***	0.686	0.524	0.643	0.449	0.584	0.429	0.701	0.338	0.700	0.543	0.649	0.481	0.813	0.527	0.714	0.556	0.798	0.560	0.667	0.429	0.700	0.471	0.700	0.386	0.810	0.512	0.560	0.488	0.777	0.536
	(0.295)	(0.304)	(0.477)	(0.347)	(0.519)	(0.250)	(0.399)	(0.194)	(0.469)	(0.223)	(0.275)	(0.291)	(0.428)	(0.348)	(0.328)	(0.253)	(0.511)	(0.325)	(0.493)	(0.186)	(0.351)	(0.350)	(0.379)	(0.227)	(0.513)	(0.257)	(0.359)	(0.274)	(0.411)	(0.322)
***−s***	0.733	0.381	0.786	0.429	0.532	0.299	0.792	0.403	0.600	0.500	0.610	0.481	0.868	0.407	0.690	0.341	1.036	0.452	0.571	0.508	0.671	0.529	0.886	0.386	0.869	0.452	0.762	0.440	0.875	0.446
	(0.240)	(0.233)	(0.378)	(0.226)	(0.231)	(0.227)	(0.552)	(0.262)	(0.476)	(0.238)	(0.136)	(0.180)	(0.416)	(0.242)	(0.422)	(0.209)	(0.652)	(0.235)	(0.323)	(0.373)	(0.541)	(0.263)	(0.710)	(0.324)	(0.280)	(0.288)	(0.374)	(0.267)	(0.563)	(0.215)
***+f***	0.000	0.019	0.000	0.000	0.000	0.013	0.000	0.000	0.000	0.029	0.000	0.000	0.022	0.000	0.000	0.008	0.000	0.024	0.000	0.000	0.029	0.043	0.000	0.029	0.000	0.000	0.000	0.012	0.000	0.018
	(0.000)	(0.050)	(0.000)	(0.000)	(0.000)	(0.034)	(0.000)	(0.000)	(0.000)	(0.076)	(0.000)	(0.000)	(0.058)	(0.000)	(0.000)	(0.021)	(0.000)	(0.041)	(0.000)	(0.000)	(0.076)	(0.079)	(0.000)	(0.076)	(0.000)	(0.000)	(0.000)	(0.031)	(0.000)	(0.030)
***−f***	0.000	0.029	0.000	0.020	0.013	0.000	0.000	0.013	0.014	0.014	0.026	0.065	0.011	0.033	0.000	0.024	0.012	0.012	0.016	0.016	0.000	0.057	0.014	0.043	0.000	0.024	0.012	0.024	0.027	0.063
	(0.000)	(0.052)	(0.000)	(0.054)	(0.034)	(0.000)	(0.000)	(0.034)	(0.038)	(0.038)	(0.044)	(0.069)	(0.029)	(0.061)	(0.000)	(0.030)	(0.031)	(0.031)	(0.042)	(0.042)	(0.000)	(0.053)	(0.038)	(0.053)	(0.000)	(0.041)	(0.031)	(0.041)	(0.033)	(0.072)
***+v***	0.048	0.048	0.020	0.010	0.013	0.104	0.000	0.039	0.043	0.029	0.000	0.013	0.011	0.011	0.008	0.056	0.012	0.048	0.032	0.016	0.043	0.057	0.043	0.057	0.024	0.095	0.000	0.012	0.054	0.134
	(0.084)	(0.050)	(0.054)	(0.027)	(0.034)	(0.161)	(0.000)	(0.072)	(0.079)	(0.076)	(0.000)	(0.034)	(0.029)	(0.029)	(0.021)	(0.056)	(0.031)	(0.094)	(0.084)	(0.042)	(0.079)	(0.098)	(0.053)	(0.079)	(0.041)	(0.112)	(0.000)	(0.031)	(0.098)	(0.127)
***−v***	0.076	0.019	0.071	0.041	0.026	0.026	0.052	0.026	0.043	0.071	0.000	0.039	0.022	0.011	0.032	0.032	0.060	0.048	0.032	0.048	0.071	0.043	0.043	0.000	0.024	0.060	0.071	0.012	0.045	0.089
	(0.118)	(0.033)	(0.160)	(0.081)	(0.069)	(0.044)	(0.103)	(0.044)	(0.079)	(0.076)	(0.000)	(0.049)	(0.038)	(0.029)	(0.030)	(0.044)	(0.063)	(0.045)	(0.084)	(0.059)	(0.076)	(0.079)	(0.053)	(0.000)	(0.041)	(0.063)	(0.089)	(0.031)	(0.059)	(0.080)
**Total Landmarks**	8.171	6.486	8.388	5.857	4.636	3.312	6.143	4.987	5.514	5.414	7.701	6.623	9.143	5.275	7.262	5.373	8.690	6.238	7.016	6.079	8.314	7.300	6.543	3.914	9.214	7.143	6.976	4.655	10.438	7.134
	(4.505)	(0.964)	(4.706)	(1.159)	(2.824)	(1.036)	(3.347)	(0.709)	(2.091)	(1.589)	(2.538)	(1.029)	(6.814)	(0.605)	(4.069)	(0.878)	(4.365)	(0.794)	(3.453)	(1.205)	(3.504)	(1.266)	(4.210)	(1.049)	(5.376)	(1.182)	(5.144)	(0.657)	(7.397)	(0.835)
**PCC Scores**	73.91%	−	75.00%	−	74.73%	−	76.79%	−	69.23%	−	84.13%	−	81.63%	−	67.28%	−	76.87%	−	76.47%	−	69.05%	−	75.04%	−	72.02%	−	72.11%	−	70.95%	−
	(20.85)		(22.36)		(25.01)		(17.94)		(25.90)		(19.88)		(19.13)		(24.10)		(16.13)		(20.66)		(21.00)		(22.57)		(28.23)		(21.19)		(21.66)	

**Table 6:** Logistic regression predicting the likelihood of acoustic landmarks and gender influencing the speech productions.

Variables	B	S.E.	Wald	Sig.	Exp(B)
*Gender*	0.219	0.405	0.292	0.589	1.244
*+g*	−22.337	6.544	11.650	0.001	<0.001
*−g*	20.236	6.272	10.411	0.001	614,041,778.992
*+p*	−3.322	1.109	8.382	0.00379	0.040
*−p*	4.985	1.610	9.583	0.002	146.136
*+b*	−1.604	1.142	1.973	0.160	0.201
*−b*	1.679	1.788	0.881	0.348	5.358
*+s*	−0.592	0.812	0.424	0.515	0.589
*−s*	−2.819	0.873	10.423	0.001	0.060
*+f*	9.685	6.373	2.309	0.129	16,075.299
*−f*	8.288	5.529	2.247	0.134	3974.111
*+v*	5.784	2.802	4.260	0.039	324.982
*−v*	−0.032	2.940	0.000	0.991	1.032
Constant	1.750	0.666	6.899	0.009	5.757

## Data Availability

The data presented in this study are available at the website of the TORGO database.
